# Research Questions and Priorities for Tuberculosis: A Survey of Published Systematic Reviews and Meta-Analyses

**DOI:** 10.1371/journal.pone.0042479

**Published:** 2012-07-27

**Authors:** Ioana Nicolau, Daphne Ling, Lulu Tian, Christian Lienhardt, Madhukar Pai

**Affiliations:** 1 McGill University, Montreal, Quebec, Canada; 2 Emory University, Atlanta, Georgia, United States of America; 3 Stop TB Partnership, World Health Organization, Geneva, Switzerland; Charité, Campus Benjamin Franklin, Germany

## Abstract

**Background:**

Systematic reviews are increasingly informing policies in tuberculosis (TB) care and control. They may also be a source of questions for future research. As part of the process of developing the International Roadmap for TB Research, we did a systematic review of published systematic reviews on TB, to identify research priorities that are most frequently suggested in reviews.

**Methodology/Principal Findings:**

We searched EMBASE, MEDLINE, Web of Science, and the Cochrane Library for systematic reviews and meta-analyses on any aspect of TB published between 2005 and 2010. One reviewer extracted data and a second reviewer independently extracted data from a random subset of included studies. In total, 137 systematic reviews, with 141 research questions, were included in this review. We used the UK Health Research Classification System (HRCS) to help us classify the research questions and priorities. The three most common research topics were in the area of detection, screening and diagnosis of TB (32.6%), development and evaluation of treatments and therapeutic interventions (23.4%), and TB aetiology and risk factors (19.9%). The research priorities determined were mainly focused on the discovery and evaluation of bacteriological TB tests and drug-resistant TB tests and immunological tests. Other important topics of future research were genetic susceptibility linked to TB and disease determinants attributed to HIV/TB. Evaluation of drug treatments for TB, drug-resistant TB and HIV/TB were also frequently proposed research topics.

**Conclusions:**

Systematic reviews are a good source of key research priorities. Findings from our survey have informed the development of the International Roadmap for TB Research by the TB Research Movement.

## Introduction

Tuberculosis (TB) continues to pose a major threat to global health [1], and research is a key component of the Global Plan to Stop TB2011-2015 [2]. Research is particularly critical for developing new tools and approaches needed for eliminating TB by 2050 [3]. Recognizing this, the Stop TB Partnership and the World Health Organization's (WHO) Stop TB Department have launched the TB Research Movement, with the aim of boosting TB research and accelerating progress in TB control towards international targets [4]. One of the main outputs of the TB Research Movement in 2011 was the publication of the *International Roadmap for Tuberculosis Research* [5] in October of 2011. This roadmap outlines all priority areas for investment in TB research and is intended to promote coordination and harmonization of scientific work on TB. Research priorities are identified in the areas of epidemiology; fundamental research; R&D of new diagnostics, drugs and vaccines; and operational and public health research. The ultimate goal is to reach all populations, including people with TB/HIV co-infection or MDR-TB and children, with new and better methods of prevention, diagnosis and treatment [5].

The process for developing this roadmap has been recently described by Lienhardt and colleagues [4]. Briefly, the research roadmap was developed through a priority ranking exercise conducted by a multidisciplinary group of 50 research experts, a multidisciplinary Delphi consultation, a series of systematic reviews and an open web-based survey [4].Among the systematic reviews that were commissioned, one was focused on all the TB research agendas that have been published from 1998 to 2010 [6]. As a next step, we were commissioned to review all the published systematic reviews and meta-analyses on TB (in all areas, including drugs, vaccines, diagnostics), to assess what research priorities have been identified in these reviews. The objectives of our systematic review were as follows: (1) to identify all systematic reviews and meta-analyses pertaining to any aspect of tuberculosis from 2005 to 2010, and (2) to assess, compile and rank the research priorities that were identified.

## Methods

### Searching

MEDLINE, EMBASE, Web of Science, and the Cochrane Library were searched for systematic reviews and meta-analyses on TB. The search strategy was developed in consultation with a medical librarian. The search was limited to systematic reviews and meta-analyses published between January 1, 2005 and July 1, 2010, in order to focus on contemporary TB literature and identify research priorities of greatest relevance to current TB control.

The search strategy included the following keywords and MeSH terms: [‘tuberculosis’ (explode) OR ‘*Mycobacterium tuberculosis*’(explode) OR ‘tuberculosis’.ti,ab. OR ‘tuberculos*’.tw] AND [‘meta analysis’ (explode) OR ‘meta analyses’.ti,ab OR ‘meta-analyses’.ti,ab OR ‘meta-analysis’.ti,ab OR ‘metanalys*’.ti,ab OR ‘systematic review’.tw]. The search was not limited to English and the last search was performed on August 18, 2010.

### Selection

Studies were included if they focused on any aspect of tuberculosis. We included systematic reviews and meta-analyses published in English, French, Spanish, and Italian. The languages included were based on the skill set of our research team. We included systematic review and meta-analyses that had focused on tuberculosis or on a tuberculosis related topic (e.g. BCG), in the title or abstract. We considered a study to be a systematic review or meta-analysis if the authors identified the study as such, or if the title or abstract contained the words “systematic review” or “meta-analysis”. Moreover, studies were regarded as systematic reviews if the authors reported a systematic, explicit approach to identify, select, and synthesize the available evidence.

The first screening of the titles and abstracts obtained following the electronic search was done by one reviewer (IN). Subsequently, the same reviewer (IN) screened the full text articles, determined the eligibility, and decided on the final inclusion of studies in the systematic review. Further, a second reviewer (MP) independently searched, screened and identified studies for the inclusion in the review.

### Data abstraction

We developed a data extraction form which was pilot-tested by two reviewers (IN and DL). The reviewers independently piloted the forms until there were no major disagreements in the data extraction process. One reviewer (IN) extracted the data from all the included studies and the second reviewer (DL) extracted data in duplicate for a random subset of 15% of the total number of included articles. Additionally, a third reviewer (LT) independently extracted data for all included studies on the study characteristics section of the data extraction form. Disagreements between the three reviewers were resolved by consensus.

### Study characteristics

We extracted data from the text or online supplement of each included systematic review or meta-analysis. Information was collected on two main points: i) the main focus of the systematic review, and ii) questions and priorities identified for future research. The UK Health Research Classification System (HRCS) [7], developed by the UK Clinical Research Collaboration for the classification and analysis of all types of health research, was used to determine the focus of the included studies as well as the focus of the research questions/priorities. In particular, the HRCS Research Activity Codes [7] were used to assign a category for the main focus of the studies and the research questions/priorities.

The main focus of each included systematic review was determined by extracting keywords from the title and abstract and matching them with the criteria developed by the HRCS. The Codes were divided into eight major categories: (1) Underpinning research; (2) Aetiology; (3) Prevention of disease and conditions, and promotion of well-being; (4) Detection, screening and diagnosis; (5) Development of treatments and therapeutic interventions; (6) Evaluation of treatments and therapeutic interventions; (7) Management of diseases and conditions; and (8) Health and social care services research (see Table 1 for full description). These research categories were used in Tables 2 and 3, to provide an overarching framework for grouping TB research.

**Table 1:** Description of the Health Research Classification System.

Research Activity Code (*Description*) and Subcategory
**1. Underpinning research: (** ***Research that underpins investigations into the cause, development, detection, treatment and management of diseases, conditions and ill health.*** **)**
1.1 Normal biological development and functioning (i.e. genes, molecular and biological pathways)
1.2 Psychological and socioeconomic processes (i.e. health and well-being)
1.3 Chemical and physical sciences (i.e. molecular modeling, chemical structures, bioengineering)
1.4 Methodologies and measurements (i.e. statistical methods, mapping methodologies, biological/socioeconomic research methods)
1.5 Resources and infrastructure (i.e. development/distribution of resources, cell lines, DNA banks, infrastructure to support research networks and centers)
**2. Aetiology: (** ***Identification of determinants that are involved in the cause, risk or development of diseases, conditions and ill health.*** **)**
2.1 Biological and endogenous factors (i.e. risk factors liked to ethnicity, age, gender, gene products)
2.2 Factors relating to physical environment (i.e. physical agents, environmental surroundings, radiation and pollution)
2.3 Psychological, social, and economic factors (i.e. individual or group behaviors and lifestyle)
2.4 Surveillance and distribution
2.5 Research design and methodologies-under aetiology category (i.e. development and evaluation of novel research designs, new epidemiological research measurements)
2.6 Resources and infrastructure (under aetiology category)
**3. Prevention of disease and conditions, and promotion of well-being: (** ***Research aimed at the primary prevention of disease, conditions or ill health, or promotion of well-being.*** **)**
3.1 Primary prevention interventions to modify behaviors or promote well-being (i.e. risk behaviors associated with diet, tobacco use, alcohol, substance misuse)
3.2 Interventions to alter physical and biological environmental risks (i.e. radiation, second hand smoke, physical and chemical agents)
3.3 Nutrition and chemoprevention
3.4 Vaccines
3.5 Resources and infrastructure (prevention)
**4. Detection, screening and diagnosis: (** ***Discovery, development and evaluation of diagnostic, prognostic and predictive markers and technologies.*** **)**
4.1 Discovery and preclinical testing of markers and technologies
4.2 Evaluation of markers and technologies
4.3 Influences and impact of screening and factors affecting uptake (i.e. attitudes and beliefs such as culture and religious practices, issues relating to gender/age/ethnicity, genetic counseling)
4.4 Population screening programmes (i.e. feasibility studies, evaluation of effectiveness, benefits and economic evaluation)
4.5 Resources and infrastructure (detection)
**5. Development of treatments and therapeutic interventions: (** ***Discovery and development of therapeutic interventions and testing in model systems and preclinical settings.*** **)**
5.1 Identification and development of pharmaceuticals (i.e. drug screening, mechanism of action, pharmacogenetics)
5.2 Discovery and development of cellular, tissue and gene therapies (i.e. gene therapy, stem cell therapy, development of delivery systems)
5.3 Discovery and development of medical devices
5.4 Development of surgical interventions
5.5 Radiotherapy
5.6 Resources and infrastructure (development of treatments)
**6. Evaluation of treatments and therapeutic interventions: (** ***Testing and evaluation of therapeutic interventions in clinical, community or applied settings.*** **)**
6.1 Pharmaceuticals (i.e. phase I, II, III and IV trials)
6.2 Clinical application and evaluation of cellular, tissue and gene therapies
6.3 Clinical and applied application and evaluation of surgical interventions
6.4 Radiotherapy
6.5 Resources and infrastructure (evaluation of treatments)
**7. Management of diseases and conditions: (** ***Research into individual care needs and management of disease, and conditions or ill health.*** **)**
7.1 Studies of patients and service user care needs
7.2 Studies involving all issues related to palliative care and end of life care
7.3 Management of diseases, ill health and conditions by health and social care professionals
7.4 Resources and infrastructure (disease management)
**8. Health and social care services research: (** ***Research into the provision and delivery of health and social care services, health policy and studies of research design, measurements and methodologies.*** **)**
8.1 Examining the organization and provision of health and social care services and evaluating factors affecting the quality of care
8.2 Economic evaluation of health and social care interventions and delivery
8.3 Policy, ethics and research governance
8.4 Development of research designs and novel methodologies for health care including treatment, management and health services research
8.5 Resources and infrastructure (health services)

**Table 2:** Focus of tuberculosis systematic reviews.

Category and subdivision	TB research focus	Proportion (%)
**Aetiology: 28 of N = 141** a **(19.9%)**		
Biological and endogenous risk factors	Genetic susceptibility/gene targets; risk factors for MDR-TB; vitamin D receptor	11/28 (39.3)
Factors relating to the physical environment	Travel risk and LTBI; nosocomial exposure to TB	5/28 (17.9)
Socio-economic risk factors	Risk of TB transmission	1/28 (3.6)
Surveillance and distribution	TB/HIV; MDRTB and HIV; diabetes and TB	11/28 (39.3)
**Prevention of disease and conditions, and promotion of well-being: 17 of N = 141 (12.1%)**		
Intervention to modify risk behaviours and lifestyles	Tobacco	3/17 (17.6)
	Alcohol	2/17 (11.8)
	Diet/BMI	1/17 (5.9)
Interventions to alter physical and biological environment	Air pollutant	1/17 (5.9)
Nutrition and chemoprevention	Isoniazid Preventive Therapy in MDRTB	2/17 (11.8)
Vaccines		5/17 (29.4)
**Detection, screening and diagnosis: 46 of N = 141 (32.6%)**		
Discovery and preclinical testing of technologies	Non-pulmonary TB	1/46 (2.2)
Evaluation of markers and technologies	Bacteriological TB diagnostic tests	17/46 (37.0)
	Bacteriological MDRTB diagnostic tests	9/46 (19.6)
	Immunological diagnostics	9/46 (19.6)
	LTBI diagnostic tests	3/46 (6.5)
Population surveillance	Active case finding TB, TB/HIV	3/46 (6.5)
	TB screening	1/46 (2.2)
**Development and evaluation of treatments and therapeutic interventions** b **: 33 of N = 141 (23.4%)**		
Pharmaceuticals	Drug-resistant TB	10/33 (30.3)
	TB treatment	9/33 (27.3)
	LTBI treatment	6/33 (18.2)
	TB/HIV treatment	3/33 (9.1)
Surgery	Spinal TB	1/33 (3.0)
Radiotherapy	Laser therapy for TB	1/33 (3.0)
**Management of diseases/condition: 14 of N = 141 (9.9%)**		
Individual care needs, secondary disease prevention	TB treatment adherence; reminder systems for LTBI treatment	7/14 (50.0)
Organization and delivery of programs, and factors affecting quality of care	DOT program evaluation; cost-benefit analysis of TB health services; evaluating quality of care	7/14 (50.0)

a Denominator N = 141 represents the total number of research focuses identified by all the included reviews. In this case N is greater than the 137 number of included systematic reviews because some reviews had a research focus captured by more than one category.

b Categories 5 and 6 of Table 1 were merged. Note: Cells do not equal 100% because the subdivisions “Other” in “Prevention of diseases and conditions”, “Detection, screening, and diagnosis”, and “Development and evaluation of treatments” were omitted. There was no SR on “Underpinning research” and “Health and social services research.”

**Table 3:** Summary of research priorities identified.

Category and subdivision	TB research priority identified	Proportion (%)
**Underpinning research: 6 of N = 191** c **(3.1%)**		
Biological pathways and processes	Investigating pathways risk of infection and infection to disease; detecting mechanism TB influences lung cancer	2/6 (33.3)
Methodologies and measurements	Implementing large scale studies for precise estimate of the epidemic status of TB/HIV co-infection.Defining immune reconstitution inflammatory syndrome; developing multicentric studies to form a criteria that differentiate pericardial TB	4/6 (66.7)
**Aetiology: 42 of N = 191 (22.0%)**		
Biological and endogenous risk factors	Detecting genetic susceptibility/gene targets/TB gene clusters; investigating biological risk factors HIV/TB and XDRTB; determining BMI and diet risk factors for TB	15/42 (35.7)
Factors relating to the physical environment	Investigating travel risk for LTBI, and the risk of air travel and fuel combustion/air pollutants for TB	7/42 (16.7)
Socio-economic risk factors	Investigating the risk of TB transmission in asylum seekers/refugees/immigrants	2/42 (4.8)
Surveillance and distribution	Implementing large scale studies to better diagnose and survey TB/HIV co-morbidity and epidemic status	1/42 (2.4)
Research design and methodologies	Designing well-powered studies to measure HIV status and drug resistance in TB patients; designing blinded, prospective studies to compare fluorescence to conventional microscopy, and sputum processing methods to direct smears in high and low HIV prevalence settings; innovating designs for studies on phage assays; creating reference standards and laboratory protocols for evaluating NAATs; improving methodology on quality appraisal	17/42 (40.5)
**Prevention of disease and conditions, and promotion of well-being 15 of N = 191 (7.9%)**		
Intervention to modify risk behaviours and lifestyles	Evaluating the association between tobacco/passive smoking/biomass fuel combustion and increase TB risk	4/15 (26.7)
	Investigating the association between alcohol use and TB risk	1/15 (6.7)
	Investigating diet/BMI/diabetes mellitus as TB risk factors	1/15 (6.7)
Nutrition and chemoprevention	Assessing the effects of isoniazid preventive therapy and risk of monoresistance in TB	1/15 (6.7)
	Investigating the relationship between vitamin D supplements/nutritional supplements and TB	2/15 (13.3)
Vaccines	Developing vaccine candidates and protective markers	6/15 (40.0)
**Detection, screening and diagnosis: 50 of N = 191 (26.2%)**		
Discovery and preclinical testing of technologies	Developing bacteriological TB diagnostic tests	5/50 (10.2)
	Bacteriological MDRTB diagnostic tests	5/50 (10.2)
	Discovering new immunological diagnostics	3/50 (6.0)
	Developing LTBI diagnostic tests	1/50 (2.0)
	Other	1/50 (2.0)
Evaluation of markers and technologies	Evaluating bacteriological TB diagnostic tests	14/50 (28.0)
	Evaluating bacteriological MDRTB diagnostic tests	4/50 (8.0)
	Evaluating new immunological diagnostics	6/50 (12.0)
	Evaluating new LTBI diagnostic tests	1/50 (2.0)
	Other	4/50 (8.0)
Influences and impact	Assessing the contribution of diagnostic tests to health care systems; factors affecting uptake such as economic and social factors	3/50 (6.0)
Population surveillance	Intensifying active case finding as a method to control TB	2/50 (4.0)
	Evaluating the usefulness of TB screening in health care workers	1/50 (2.0)
**Development of treatment regimens and therapeutic interventions: 11 of N = 191 (5.8%)**		
Pharmaceuticals	Developing treatment for active TB	3/11 (27.3)
	Discovering treatment for drug-resistant TB	3/11 (27.3)
	Developing TB/HIV treatment	5/11 (45.4)
**Evaluation of treatments and therapeutic interventions: 37 of N = 191 (19.4%)**		
Pharmaceuticals	Evaluating treatments for TB	8/37 (21.6)
	Evaluating treatments for drug-resistant TB	11/37 (29.7)
	Evaluating TB/HIV treatments	12/37 (32.4)
	Other	1/37 (2.7)
Surgery		2/37 (3.1)
Other		3/37 (8.1)
**Management of diseases/condition**		
Individual care needs, secondary disease prevention	Assessing TB treatment adherence; reminder systems for LTBI treatment	13/191 (6.8)
**Health and social care services research**		
Organization and delivery of programs, and factors affecting quality of care	Evaluating DOT program; cost-benefit analysis of TB health services; evaluating quality of care	16/191 (8.4)
**Other**		1/191 (0.5)

c Denominator N = 191 represents the total number of research priorities identified by all the included studies.

In the HRCS, each of the eight major categories is further subdivided into five to nine subcategories with definitions for the type of research that belonged to that subcategory. For instance, “(1)Underpinning research” includes five subcategories: (1.1)studies of normal biological development and functioning, including gene, gene products, biological pathways, molecular and cellular structures, and development and characterization of model systems; (1.2) studies that do not address health directly but cover issues such as psychological and socioeconomic processes, individual or group characteristics and behaviours, and social and cultural beliefs; (1.3) research in chemical and physical sciences that may lead to the future development of diagnostic tools or treatments; (1.4) studies that target the development of novel methodologies and measurements including the development of statistical methods, and the development of mapping methodologies; and (1.5)research involving the development and/or distribution of resources for use by the research community, and infrastructure to support research networks. Using the main categories and the subdivisions within each category, we mapped the corresponding TB research areas found in the literature search (refer to Tables 1 and 2).

### Quantitative data synthesis

Study characteristics were summarized using descriptive statistics. Measures such as total count, frequency, and proportion, were used to summarize data. Data analyses were performed using STATA Version 11.0.

## Results

**Figure 1:**
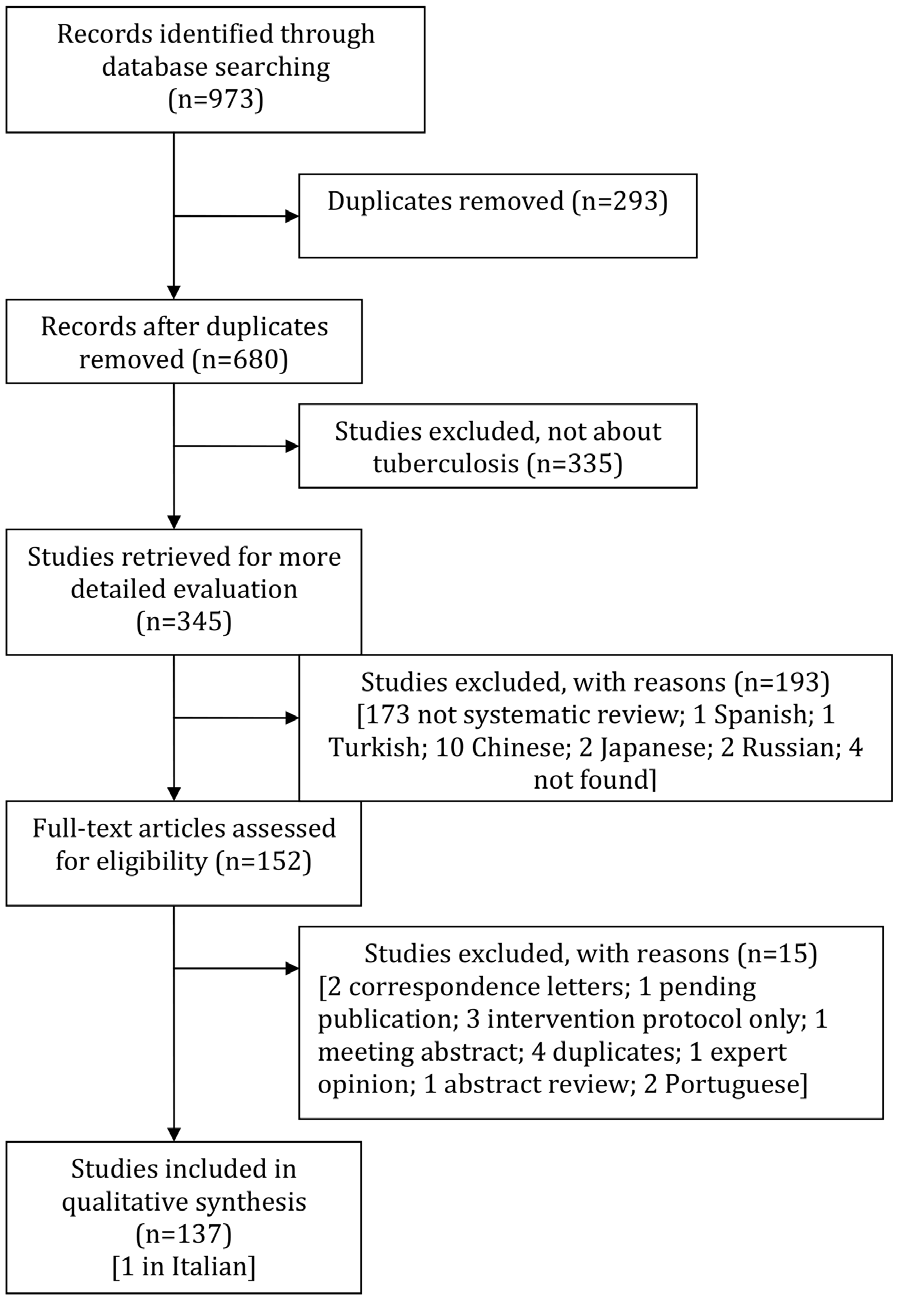
Flow chart of the study selection process.

There were a total of 973 records identified through the electronic database search (Figure 1). The first screening of titles and abstracts was done on 680 records. Following the first screening process, 528 records were excluded. The reasons for exclusion are listed in Figure 1. The full text screening of articles was performed on 152 records. Overall, there were 137 systematic reviews included in our analysis [8]–[144].

### Characteristics of included TB systematic reviews

The 137 reviews were published in 61 different journals. The majority of reviews (39.4%) were published in journals with impact factors of five or less, and only six (4.3%) reviews were published in journals with a high impact factor (>15). However, a large proportion of the reviews (38.6%) were published in journals that did not have an impact factor. In addition, approximately 24% of the main authors were from the United States and 41% were from four other countries (China, UK, Canada, and Italy). The remaining 34.1% of authors were from 26 different countries.

Out of the 137 reviews, 131 (95.6%) self identified as a systematic review or meta-analysis, which means that they used the term “systematic review” or “meta-analysis” in the title or abstract. Approximately 91% (124) of all reviews were not Cochrane reviews. Among the 13 Cochrane reviews, 9 of them focused on “evaluation of treatments and therapeutic interventions”.

Half of the reviews (67 [48.9%]) reported having a funding source, whereas only 15 reviews (11.0%) reported not being funded and 55 reviews (40.1%) did not report funding status. Most of the reviews (109 [79.6%]) included less than 50 studies in their review and within those reviews, the majority had between 1,000 and 10,000 participants (34/109[31.2%]).

### Focus of TB systematic reviews

The main focus of each review was determined using the HRCS as described in the Methods section. The classification categories were subdivided into major tuberculosis research areas as described in Table 2. The three most common review categories, in decreasing order, were “Detection, screening and diagnosis” with 46/141(32.6%) systematic reviews, “Development and evaluation of treatments and therapeutic interventions” with 33/141(23.4%) systematic reviews and “Aetiology” with 28/141(19.9%) systematic reviews.

Within the category of “Detection, screening and diagnosis”, 17/46 (37%) of the reviews focused on bacteriological diagnostics for active TB, such as improving processing methods of sputum smear microscopy, and assessing the use of nucleic acid amplification tests (NAATs). The two other most common TB research aims were bacteriological diagnostics for MDR-TB (9/46[20%]) and immunological diagnostics (9/46[20%]). More specifically, bacteriological diagnostics for MDR-TB included tests such as line-probe assays, bacteriophage based assays, and colorimetric redox assays. Immunological diagnostics were focused mainly on testing and evaluating interferon-gamma release assays (IGRAs).

In the category “Development and evaluation of treatments and therapeutic interventions”, 10/33 (30%) studies focused on drug resistant tuberculosis treatment, 9/33 (27.3%) studies on evaluating different regimen combinations for tuberculosis treatment, and 6/33(18.2%) on treatment of latent tuberculosis infection (LTBI).

In the category “Aetiology”, 11/28 (39.3%) systematic reviews focused on biological/genetic risk factors such as genetic susceptibility and gene targets,11/28 (39.3%) studies targeted surveillance and distribution of TB/HIV co-infection, MDRTB and HIV, and diabetes and TB, and 5/28(17.9%) focused on travel risk for LTBI and nosocomial TB exposure.

### Research priorities

Out of 137 reviews, 103 (75%) identified at least one research question or a research priority. Of these, 48 (46.6%) identified only one research priority, 33 (32.0%) two research priorities, 7 (6.8%) three, 7 (6.8%) four, and 8 (7.8%) five research priorities. None of the reviews identified more than five research priorities.

Table 3 shows the summary of research priorities by category, subdivision, and TB-specific research priority. The three major categories of research priorities/questions were “Detection, screening and diagnosis” responsible for 50/191 (26.2%) of all the identified research priorities, “Aetiology” with 42/191 (22.0%), and “Evaluation of treatments and therapeutic interventions” with 37/191 (19.4%).

In the most common category, “Detection, screening and diagnosis”, the top research priority was the evaluation of bacteriological TB diagnostic tests in 14/50 (28.0%) reviews. Other frequently cited TB research priorities were: evaluation of immunological TB diagnostic tests (6/50 [12.0%]); discovery and development of new TB diagnostic tests (5/50 [10.2%]); and development of new bacteriological MDR-TB diagnostics (5/50 [10.2%]). Two priorities had almost equal importance and were highly prevalent in TB literature. The main priority in that category was to investigate the detection, screening and diagnosis of drug-resistant TB and MDR-TB. Studies called for the need to develop studies that detect resistance from smear positive specimens, determine the accuracy of colorimetric methods, line-probe assays, phage-based assays for rapid screening and nitrate reductase assay (NRA), and find the clinical usefulness of rapid diagnosis of rifampicin-resistant TB. Another frequency priority was to address unresolved research questions on interferon-gamma release assays (IGRAs), discover new antigens with immunodiagnostic potential, and test IGRAs in various populations and settings to establish test reproducibility. Evaluating sputum processing methods and smear microscopy, assessing nucleic acid amplification tests (NAATs), and evaluating tests for extrapulmonary TB (e.g. adenosine deaminase for pleural TB) were commonly cited priorities.

Within the “Aetiology” category, the main TB research priorities were: development of new research methods; better study designs or statistical tools for studying drug resistant TB, MDR-TB, links between HIV and MDR-TB; comparison of diagnostic tests (17/42 [40.5%]); identification of biological and genetic risk factors (15/42 [35.7%]); and evaluation of the role of risk factors such as tobacco and air pollutants (7/42 [16.7%]). The most frequent priority was to examine gene and gene products in relation to TB disease and susceptibility to disease. Key genes such as vitamin D receptor polymorphisms, IL10 gene, and drug-metabolizing enzyme (DME) gene polymorphisms were commonly mentioned for future research. The second most frequent research priority on TB/HIV included recommendations to conduct studies investigating XDR-TB and HIV co-infection, identifying a comprehensive definition of IRIS (immune reconstitution inflammatory syndrome), and investigating sputum processing methods with direct smears in settings with high and low HIV prevalence.

The category “Evaluation of treatments and therapeutic interventions” was the third most frequent. It focused on TB/HIV drug treatments (12/37 [32.4%]), drug-resistant TB treatments (11/37 [29.7%]), new TB drugs and active tuberculosis regimens (8/37[21.6%]). Implementing studies that evaluate new treatments and therapeutic interventions for drug-resistant TB, MDR-TB, and XDR-TB, was a prominent research priority. Such studies would need to examine methods to improve treatment outcomes for patients with XDR TB such as using later-generation fluoroquinolones, discovering methods to tailor treatment regimens for various forms of TB drug resistance, and investigating the use of quality-controlled laboratory testing for all first and second-line drugs that define XDR-TB. Another frequently cited priority was designing trials to evaluate the optimal duration of TB treatment, the influence of level of immunosuppression on effectiveness of TB drugs, and the combination of anti-TB chemoprophylaxis with antiretroviral therapy.

## Discussion

Systematic reviews and meta-analyses are widely acknowledged as a key component of the policy and guideline development process [145]. A large number of systematic reviews have been published in the area of TB diagnostics [146], and these are increasingly being used for developing guidelines [147]. To this end, the Grading of Recommendations Assessment, Development and Evaluation (GRADE) tool has increasingly been adopted by policy makers and guideline developers to provide an explicit, comprehensive and transparent process for moving from evidence to recommendations [145].

Systematic reviews often conclude by making suggestions for the direction of future research, and thus could be a good source for identifying the most important questions for TB research. Our survey collected descriptive information from all eligible systematic reviews and meta-analyses that were subsequently used to generate a list of research priorities in TB which were used for developing the International Roadmap for Tuberculosis Research [5].

Our systematic search showed that a fairly high number of systematic reviews were published on TB during the period of 2005 to 2010. The findings of our review need to be interpreted along with a recent systematic review by Rylance and colleagues [6] on 33 articles with research agendas on TB. These authors found that the top priority areas for research were new TB drug development (28 articles), diagnosis and diagnostic tests (27), epidemiology (20), health services research (16), basic research (13), and vaccine development and use (13).

In our review of 137 TB systematic reviews, the top three categories for the focus of the research priorities/questions were “Detection, screening and diagnosis” “Aetiology” and “Evaluation of treatments and therapeutic interventions.” TB diagnosis and treatment were among the most important research priorities in both reviews. One possible reason of why TB diagnosis research ranked high on our list could be that our review focused on years 2005 to 2010, a period when major advances have been made in TB diagnostics, especially with IGRAs becoming a very popular subject of research [148]. Also, this time period saw the introduction of several WHO policies on TB diagnostics. Further, the emphasis on new tools in the Global Plan to Stop TB 2006–2015 [149], along with the creation of product development partnerships such as the Foundation for Innovative New Diagnostics (FIND), AERAS, and Global Alliance for TB Drug Development, may have inspired research on new diagnostics and drugs.

The research priorities determined were mainly focused on the discovery and evaluation of bacteriological TB tests, drug-resistant TB tests and immunological tests, with special focus on IGRA tests. Also, tests for extra-pulmonary TB came up as a frequently cited priority in the Detection of TB category. Other important topics of future research were genetic susceptibility to TB and disease determinants attributed to HIV/TB. Evaluation of drug treatments for TB, drug-resistant TB and HIV/TB were also frequently proposed research topics. Many articles cited the need for improved and tailored treatment methods for MDR-TB and XDR-TB.

Although several systematic reviews identified areas for further research, the questions themselves were often framed in a generic way, rather than in a highly focused manner with specific recommendation for action. Future TB systematic reviews will need to be more focused, and propose highly specific, answerable questions that are amenable to well-designed research studies.

Our study has several limitations. Due to the poor overall quality of reporting of the systematic reviews, the findings may not be representative of the general output from the TB research community [150]. The inclusion of eligible studies was limited by the fact that we only reviewed articles in three other languages besides English. We were also unable to search ‘grey’ literature, contact authors, or hand search journals. The review also did not include any unpublished literature. Due to its overarching and generic nature, the Health Research Classification System categories were at times non-specific and difficult to match with specific areas of TB research. Furthermore, it was difficult to classify research priorities into narrow subdivisions since some research priorities could qualify for more than one subdivision. By categorizing research priorities into larger, predefined categories, we lost detailed information on individual research priorities. To remedy this, we condensed each priority and extracted the topic words from it. The topic words were then grouped together to form the summary of repeated priorities/questions and calculate the frequency.

There has been a lot of recent attention and focus on childhood TB, but because our search was last performed in 2010, our analysis may have missed research priorities in this important area.

In summary, our systematic review of published systematic reviews on TB helped identify several key priorities for future TB research. This exercise was useful to describe the landscape of TB research and the overarching TB research themes arising from systematic reviews and meta-analyses conducted over the last 5 years. Their scope is, however, limited, since systematic reviews themselves are influenced by current hot topics or new technologies. They are nevertheless useful in indicating research priorities on areas that receive high attention, either due to recent scientific developments or increasing questions surrounding advancement of knowledge in these very areas. They bring useful additional arguments and information to the broader, deeper and more rigorously conducted process of international research agenda development.
